# Molecular regulatory mechanisms of osteoclastogenesis through cytoprotective enzymes

**DOI:** 10.1016/j.redox.2016.01.006

**Published:** 2016-01-11

**Authors:** Hiroyuki Kanzaki, Fumiaki Shinohara, Itohiya Kanako, Yuuki Yamaguchi, Sari Fukaya, Yutaka Miyamoto, Satoshi Wada, Yoshiki Nakamura

**Affiliations:** a Tohoku University Hospital, Maxillo-Oral Disorders, Japan; b Department of orthodontics, School of Dental Medicine, Tsurumi University, Japan; c Tohoku University Graduate School of Dentistry, Oral Microbiology, Japan

**Keywords:** Osteoclast, Oxidative stress, Heme-oxygenase 1 (HO-1), Nrf2, FOXO, Sirtuin

## Abstract

It has been reported that reactive oxygen species (ROS), such as hydrogen peroxide and superoxide, take part in osteoclast differentiation as intra-cellular signaling molecules. The current assumed signaling cascade from RANK to ROS production is RANK, TRAF6, Rac1, and then Nox. The target molecules of ROS in RANKL signaling remain unclear; however, several reports support the theory that NF-κB signaling could be the crucial downstream signaling molecule of RANKL-mediated ROS signaling. Furthermore, ROS exert cytotoxic effects such as peroxidation of lipids and phospholipids and oxidative damage to proteins and DNA. Therefore, cells have several protective mechanisms against oxidative stressors that mainly induce cytoprotective enzymes and ROS scavenging. Three well-known mechanisms regulate cytoprotective enzymes including Nrf2-, FOXO-, and sirtuin-dependent mechanisms. Several reports have indicated a crosslink between FOXO- and sirtuin-dependent regulatory mechanisms. The agonists against the regulatory mechanisms are reported to induce these cytoprotective enzymes successfully. Some of them inhibit osteoclast differentiation and bone destruction via attenuation of intracellular ROS signaling. In this review article, we discuss the above topics and summarize the current information available on the relationship between cytoprotective enzymes and osteoclastogenesis.

## Highlights

• Three essential regulatory mechanism for cytoprotective enzymes were reported.
• They are Nrf2, SIRT, and FOXO.
• Intracellular ROS is used as signaling molecule during osteoclastogenesis.
• Cytoprotective enzymes attenuates osteoclastogenesis by ROS scavenging.
• Nrf2, SIRT, and FOXO could be therapeutic target for bone destructive disease.

## Graphical abstract

**Figure:**
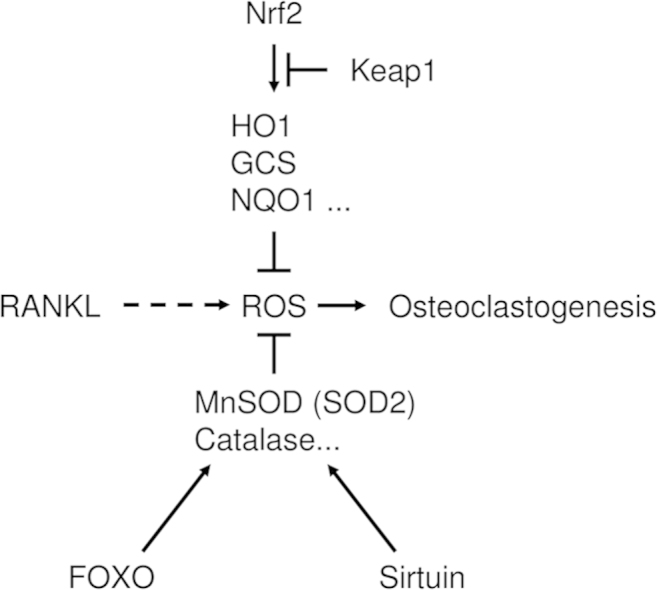

## 1 Introduction

Osteoclasts are multi-nucleated cells that resorb bone tissue [1] and are differentiated from macrophage–monocyte cell lines [2]. Osteoclast differentiation, namely osteoclastogenesis, is strictly regulated by receptor activator of nuclear factor kappa-B ligand (RANKL), an osteoclastogenic signaling cytokine [3]. Reactive oxygen species (ROS), such as hydrogen peroxide and superoxide, work as intracellular signaling molecules following RANKL signaling during osteoclastogenesis [4], [5], [6]. However, apart from their role as intracellular signaling molecules, ROS exert cytotoxic effects such as peroxidation of lipids and phospholipids [7], and oxidative damage to proteins and DNA [8]. Therefore, cells have several protective mechanisms against these oxidative stressors [9], [10], [11] most of which induce cytoprotective enzymes [12], [13], [14], [15], [16], [17], [18], [19], [20] and ROS scavenging. Taken together, it is thought that cytoprotective mechanisms are attenuated during osteoclastogenesis to intensify intracellular ROS signaling.

In this review article, we have summarized the relationship between osteoclastogenesis and the protective mechanisms that work against oxidative stressors.

## 2 ROS work as intracellular signaling molecules during osteoclastogenesis

RANKL is an essential cytokine in osteoclastogenesis [1], [21], [22], [23], and various intracellular signaling molecules, such as nuclear factor of activated T-cells (NFAT) [24], mitogen-activated protein kinase (MAPK) [25], [26], tumor necrosis factor receptor-associated factor (TRAF) [27], [28], c-jun N-terminal kinase (JNK) [29], Akt [30], and ROS [4], [5] have been identified. ROS are interesting molecules because not only do they work as intracellular signaling molecules, but also they increase with age or with the onset of an inflammatory state, which subsequently leads to bone destruction [31], [32], [33], [34], [35], [36], [37]. In addition, exogenous hydrogen peroxide induces osteoclastogenesis [38], signifying that oxidative stress participates in the regulation of osteoclastogenesis from both within the cytoplasm and extracellularly.

It is reported that TRAF6 plays a key linkage role in ROS production by RANKL [39]. We reported that dominant-interfering mutant form of TRAF6, significantly decreased ROS induction, although TRAF6 itself does not directly produce ROS. Rac, a functional downstream molecule and member of the Rho-GTPase subfamily, which is involved in the organization of the cytoskeleton, is a cytosolic component of NADPH oxidase (NOX) complex and responsible for the activation of NOXs [40]. The expression of a dominant-negative mutant of Rac1 blocks ROS production, signifying that Rac1 is responsible for regulating the generation of ROS during osteoclast differentiation [41]. In addition, NOXs have been reported as essential enzymes that produce ROS during osteoclast differentiation [42], [43], [44]. Taken together, the current assumed signaling cascade from RANK to ROS production is RANK, TRAF6, Rac1, and then NOX.

**Fig. 1:**
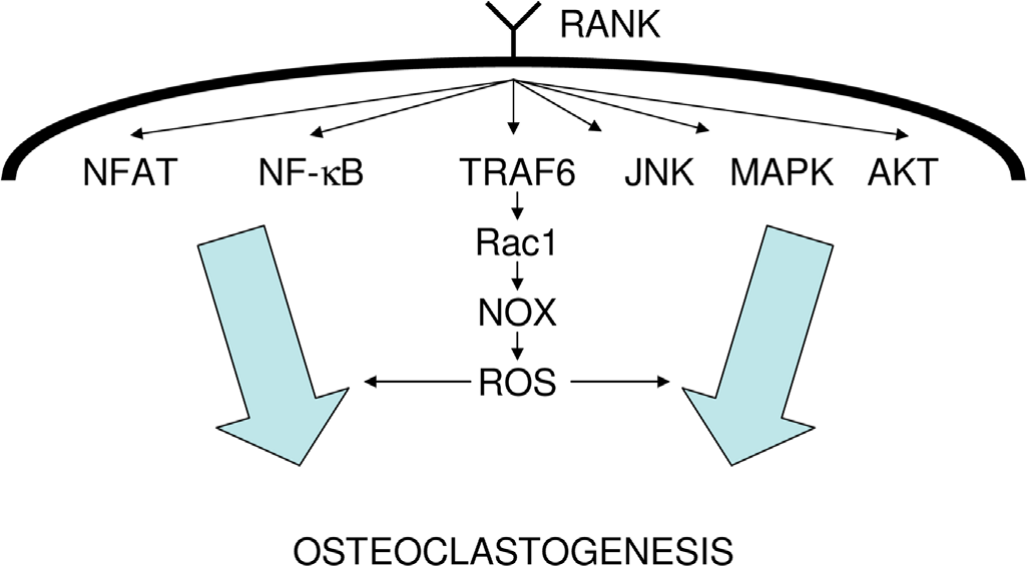
Summary of the current information about the intracellular signaling cascade of RANKL. Intracellular signaling molecules after RANK were identified. The current assumed signaling cascade from RANK to ROS production is also described. Some reports suggest that NF-κB is the crucial downstream molecule of RANKL-mediated ROS signaling.

The target molecules of ROS in RANKL signaling remain unclear; however, several reports have suggested that MAPK, PI3K, and NF-kB activation are downstream events [45], [46]. Additionally, Bharti et al. reported that curcumin, which has ROS-scavenging properties, inhibits RANKL-induced NF-κB activation, which indicates that NF-κB signaling could be the crucial downstream signaling molecule of RANKL-mediated ROS signaling [47]. Current information about the intracellular signaling cascade of RANKL is summarized in Fig. 1.

## 3 Defense mechanisms against ROS

As mentioned previously, ROS exhibit cytotoxicity [7], [8]; therefore, cells have several protective mechanisms against these oxidative stressors that mainly induce cytoprotective enzymes and ROS scavenging. The mechanisms regulating cytoprotective enzymes are summarized in Table 1.

**Fig. 2:**
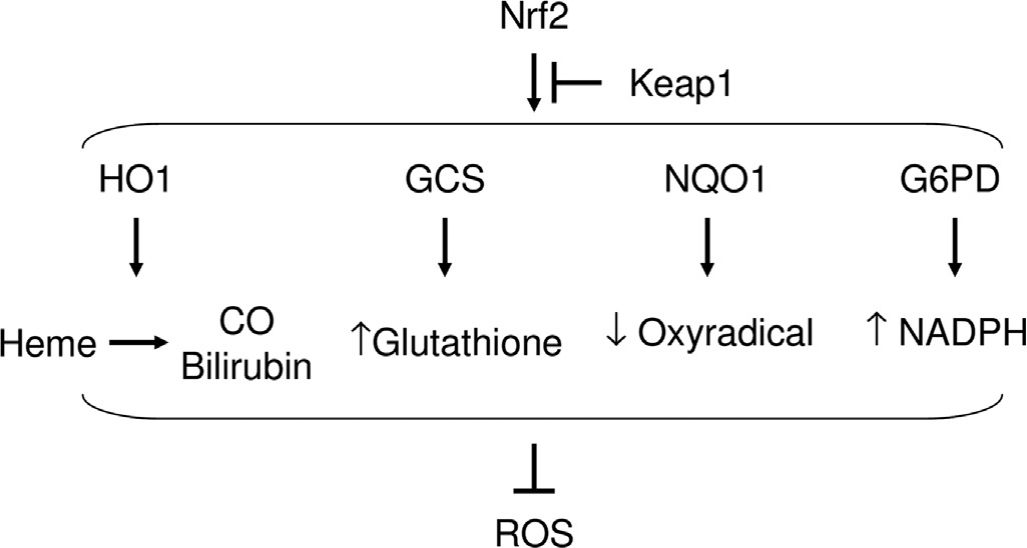
Nrf2-mediated cytoprotective enzymes scavenge ROS. Nrf2 transcriptionally regulates the expressions of HO1, GCS, NQO1, and G6PD. HO1 convert heme into carbon oxide (CO) and bilirubin, and they scavenge ROS. GCS increases intracellular glutathione, which results in ROS scavenging. NQO1 reduces oxyradicals. G6PD increases intracellular NADPH, which augments ROS scavenging.

The most renowned regulator of cytoprotective enzymes is transcriptional factor nuclear factor E2-related factor 2 (Nrf2), which controls the gene expression of many cytoprotective enzymes, such as heme oxygenase-1 (HO-1) [13], NAD (P) H: quinone reductase (NQO1) [14], gamma-glutamylcysteine synthetase (GCS) [15], and the auxiliary cellular NADPH regenerating enzyme, glucose 6-phosphate dehydrogenase (G6PD) [16] (Fig. 2); all of these enzymes are ROS scavengers [17], [18], [19], [20]. However, kelch-like ECH-associated protein 1 (Keap1) negatively regulates Nrf2-dependent transcription of cytoprotective enzymes by inhibiting nuclear translocation, cytoplasmic ubiquitination, and degradation of Nrf2 [48].

**Fig. 3:**
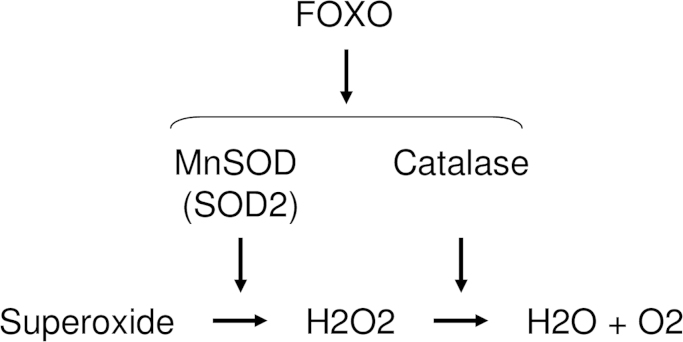
FOXO-mediated cytoprotective enzymes scavenge ROS. FOXO regulates the expressions of MnSOD (SOD2) and catalase (CAT). MnSOD convert superoxide into H2O2, followed by the conversion into H2O and O2 by CAT.

FOXO ubiquitous transcription factors, which are dephosphorylated and subsequently activated by oxidative stress, are involved in the regulation of redox balance [49], [50], [51], [52], [53]. It is reported that oxidative stress activates FOXO via mammalian Ste20-like kinases [50] and p66shc [51]. In addition, FOXO3 and 4 regulate the expression of superoxide dismutase (SOD) [51], [54] and catalase (CAT) [55], and SOD converts superoxide to hydrogen peroxide [56], which is subsequently detoxified by CAT (Fig. 3) [57]. Three isozymes of SOD—SOD1, 2, and 3—have been identified and characterized in mammals [58]. SOD1 is located in the cytoplasm, SOD2 in the mitochondria, and SOD3 is extracellular. SOD1 and SOD3 are Cu-Zn-SOD types, whereas SOD2 is Mn-SOD.

**Fig. 4:**
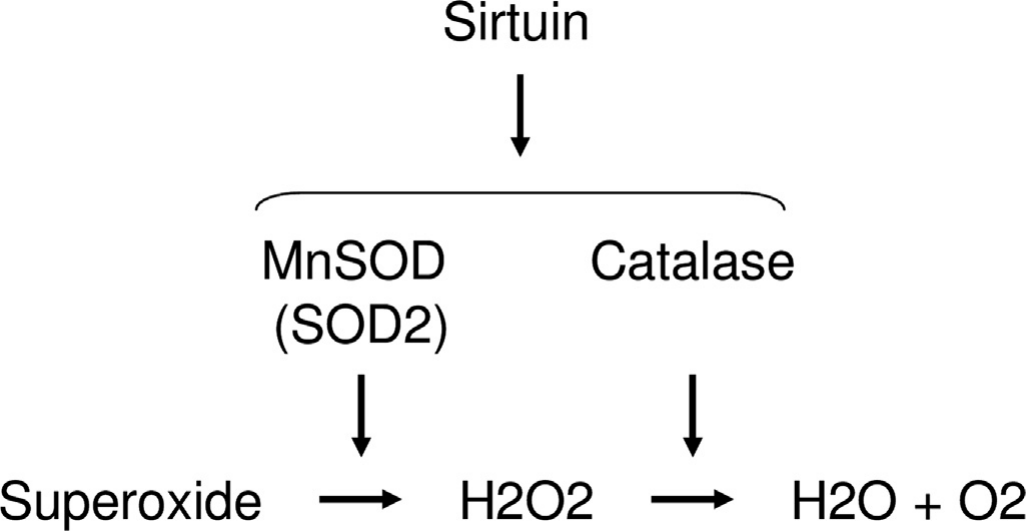
SIRT-mediated cytoprotective enzymes scavenge ROS. SIRT regulates the expressions of MnSOD (SOD2) and catalase (CAT). MnSOD convert superoxide into H2O2, followed by the conversion into H2O and O2 by CAT.

Sirtuin, which was originally identified as a protein deacetylase [59], is also a regulator of the expression of cytoprotective enzymes such as SOD [60], [61] and CAT (Fig. 4) [62]. Mammalian sirtuins consist of seven members (SIRT1–7), and have been implicated in various cellular responses including aging, transcription, apoptosis, and stress resistance [63]. Among them, the functions of Sirt1 and 3 in oxidative stress responses have been reported. SIRT1 deacetylates FOXO3 and 4 [53], which results in the upregulation of Mn-SOD [64]. Furthermore, Olmos et al. reported that SIRT1-dependent upregulation of cytoprotective enzymes depended on the formation of a FOXO3a/PGC-1α complex in vascular endothelial cells [65]. However, Chen et al. reported that SIRT3 directly upregulated SOD2 [60]. Regarding the crosslinking between SIRT and Nrf2, Huang et al. reported that SIRT1 upregulated HO-1 and SOD1 via induction of Nrf2 [66]. Overall, sirtuins, especially SIRT1 and 3, directly or indirectly regulate cytoprotective enzymes.

## 4 Cytoprotective enzymes and osteoclastogenesis

Since ROS operate as intracellular signaling molecules during osteoclastogenesis, a close relationship between osteoclastogenesis and cytoprotective enzymes is to be expected. Indeed, a well-known cytoprotective enzyme, HO-1, is a negative regulator of osteoclastogenesis [67], [68], [69]. Relationships between the mechanisms regulating cytoprotective enzymes and osteoclastogenesis have also been reported. Rana et al. reported that loss of Nrf2 accelerates ionizing radiation-induced bone loss in Nrf2 knockout mice [70]. Other groups have reported that Nrf2 negatively regulates osteoclastogenesis through attenuation of RANKL-mediated intracellular ROS signaling by cytoprotective enzymes [71], [72]. Furthermore, we previously reported that overexpression of Nrf2 induces the expression of cytoprotective enzymes, attenuates intracellular ROS signaling, and thereby inhibits osteoclastogenesis [71]. Both overexpression of Nrf2 and Nrf2 activation (induction of nuclear translocation) inhibit osteoclastogenesis [6], [73], [74]. These lines of evidence suggest that Nrf2 activation could be a therapeutic approach towards bone destructive diseases such as rheumatoid arthritis, osteoporosis, and periodontitis.

**Fig. 5:**
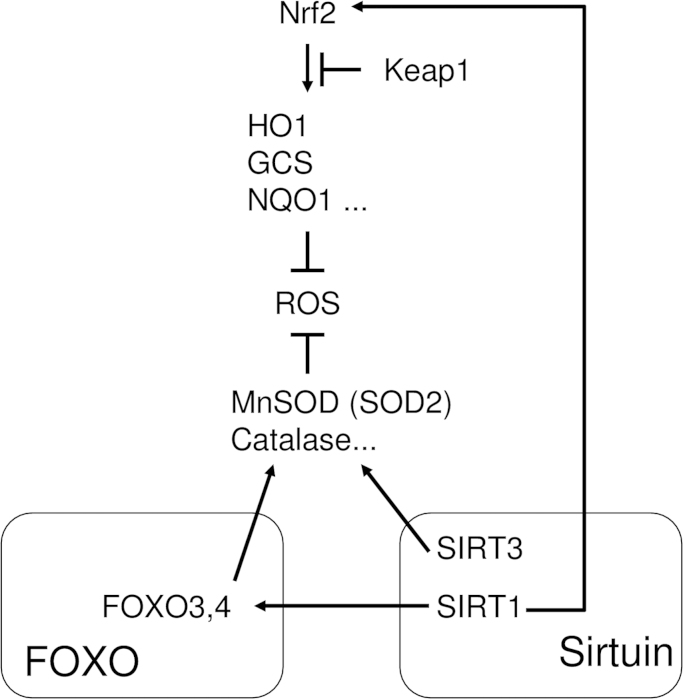
Nrf2, FOXO, and sirtuin negatively regulate osteoclastogenesis via attenuation of intracellular ROS signaling. Nrf2 regulates the transcription of cytoprotective enzymes and ROS scavenging. However, Keap1 attenuates cytoprotective enzymes via degradation of Nrf2. Other mechanisms regulating cytoprotective enzymes have roles in ROS regulation: FOXO directly, and sirtuin directly and indirectly (via FOXO).

Another mechanism regulating cytoprotective enzyme FOXO contributes to the control of osteoclastogenesis. Bartell et al. reported that FOXO protein attenuates osteoclastogenesis via augmentation of cytoprotective enzymes [75]. Sirtuins, originally identified as protein deacetylases, have been reported as suppressors of osteoclastogenesis. SIRT1 suppresses osteoclastogenesis by the upregulation of cytoprotective enzymes via FOXO-mediated transcription and subsequent attenuation of intracellular ROS signaling [76]. Lee et al. reported that the overexpression of SIRT6, an NAD (+)-dependent deacetylase, suppresses bone destruction in a collagen-induced arthritis mouse model [77]. These lines of evidence suggest that the key molecule among the mechanisms regulating cytoprotective enzymes (Nrf2, FOXO, and sirtuin) negatively regulates osteoclastogenesis via attenuation of intracellular ROS signaling (Fig. 5).

## 5 Regulatory mechanisms of potential pharmacological targets for bone destructive diseases

As discussed above, osteoclasts also possess mechanisms that regulate cytoprotective enzymes, which manage the intracellular ROS levels. Since intracellular ROS play a role in RANKL-mediated osteoclastogenesis, the mechanisms that regulate cytoprotective enzymes negatively control osteoclastogenesis via ROS scavenging mediated by cytoprotective enzymes. In other words, osteoclastogenesis is controlled via interference with the mechanisms regulating cytoprotective enzymes.

Indeed, some papers report that the activation of Nrf2 inhibits osteoclastogenesis [6], [73], [74]. The pharmacological activation of Nrf2 has been extensively explored in cancer research and chemical detoxification fields, thus potential Nrf2 activators such as sulforaphane [78], epigallocatechin gallate [79], curcumin [80], and N-acetylcysteine [81] are well-documented and known to inhibit bone destruction [72], [82], [83], [84]. Another regulatory molecule, FOXO, is also a potential therapeutic target for bone destructive diseases. Statins, HMG-CoA reductase inhibitors, induce FOXO phosphorylation [85] and exhibit osteoclastogenesis by ROS scavenging [86]. Regarding sirtuin-mediated regulatory mechanisms, resveratrol, an agonist of SIRT1 [87], inhibits osteoclastogenesis through the attenuation of ROS production [88], [89], [90]. Indeed, sirtuin activators such as resveratrol or other synthesized chemicals inhibit bone destruction [91], [92], [93], [94]. Some of the chemicals reported to activate cytoprotective enzymes and thereby inhibit bone destruction are summarized in Table 2.

## 6 Summary and perspective

In this review manuscript, we have summarized recent information about the relationship between osteoclastogenesis and the mechanisms regulating cytoprotective enzymes. Although some parts have been extensively explored, further investigations are necessary to gain a greater understanding. In particular, crosstalk among the mechanisms regulating cytoprotective enzymes and other signaling molecules should be elucidated.

Since some of the agonists that affect the mechanisms regulating cytoprotective enzymes have been reported as inhibitors of bone destruction, these chemicals could be potential drugs for the treatment for bone destructive diseases in the near future.

## Figures and Tables

**Table 1:** Regulatory mechanisms of cytoprotective enzymes.

Regulator	Cell type/experimental model	Tested function/findings	References
Nrf2	L929 fibroblast, mutant Nrf2 expression	Mutant Nrf2 decreased HO-1	[13]
	Rat NQO-1 gene, promoter assay	Nrf2 regulated NQO-1	[14]
	Human GCS gene, promoter assay	Nrf2 regulated GCS	[15]
	Nrf2 knockout mice	Nrf2 KO decreased NQO-1 and GCS	[16]

FOXO	Breast cancer cells	FOXO3 regulates MnSOD	[49]
	Primary mammalian neurons	MST-FOXO axis controls oxidative-stress response	[50]
	C. elegans and mice gene	pp66shc controls oxidative-stress response via FOXO3 (FKHRL1)	[51]
	Mutant mice	Insulin/IGF-1-FOXO pathway relates oxidative-stress Response	[52]
	Mammalian cells	Mammalian SIRT1 deacetylates FOXO3 and/or FOXO4	[53]
	Mammalian cells	FOXO3 directly increase MnSOD	[54]
	Mouse NIH3T3 cells	ROS-Ral-JNK axis mediates FOXO4-dependent MnSOD upregulation	[55]

SIRT			
	Human HEK293 cells	SIRT3 deacetylates and activates MnSOD	[60]
	Mammalian cells	SIRT3 activates MnSOD	[61]
	Renal tubular cells	SIRT1 activates catalase via FOXO3	[62]
	Mammalian cells	SIRT1 activates MnSOD via FOXO4	[64]

**Table 2:** Reported chemicals that can activate cytoprotective enzymes and thereby inhibit bone destruction.

Chemicals	References
Nrf2 activator	
Curcumin	[47], [80], [83]
EGCG	[74], [79], [82]
ETGE-peptide	[6]
Na2SO4	[73]
NAC	[81], [84]
Sulforaphane	[74], [78]
FOXO	
Statin	[86]
Sirtuin	
Resveratrol	[88], [89], [90], [91], [93]
SRT2104	[94]
SRT3025	[92]
